# Analysis of the impact of the Brazilian Suicide Prevention Campaign "Yellow September": an ecological study

**DOI:** 10.47626/2237-6089-2022-0564

**Published:** 2024-11-26

**Authors:** Walter Gabriel Neves Cruz, Thiago Aguiar Jesuino, Hercules Fernandes Moreno, Lara Garrido Santos, Amanda Galvão-de Almeida

**Affiliations:** 1 Universidade Federal da Bahia Salvador BA Brazil Universidade Federal da Bahia (UFBA), Salvador, BA, Brazil.; 2 UFBA Faculdade de Medicina da Bahia Departamento de Neurociências e Saúde Mental Salvador BA Brazil Departamento de Neurociências e Saúde Mental, Faculdade de Medicina da Bahia, UFBA, Salvador, BA, Brazil.

**Keywords:** Suicide, Brazil, mental health, preventive psychiatry, mass media

## Abstract

**Objective:**

Yellow September (YS) (Setembro Amarelo) is a Brazilian suicide prevention campaign launched in 2015, however, its effectiveness at reducing mortality is still unknown. This is an ecologically interrupted time series study that analyzed the trend in suicide rates in Brazil between 2011 and 2019 and its association with the implementation of YS at a national level.

**Methods:**

A segmented interrupted series regression analysis was performed, using a generalized linear Poisson model, with correction for seasonal trends. Data were provided by the Mortality Information System (Sistema de Informações Sobre Mortalidade [SIM]).

**Results:**

There was an increase in the annual rates of suicide deaths between 2011 and 2019, with 4.99 and 6.41 suicides per 100,000 inhabitants, respectively. The null hypothesis, that the YS did not change the historical trend of growth in suicides in Brazil after its implementation, was confirmed. However, there was a significant increase of 6.2% in the risk of mortality in 2017 and a significant increase of 8.6% in 2019.

**Conclusion:**

The results are consistent with the literature, which proposes that campaigns focused solely on publicity in the media generate unsound findings regarding the effective reduction in the number of deaths by suicide. The lack of initiatives involving multisectoral actions may explain the failure of YS to change rates of death by suicide. Therefore, implementation of new lines of action focused on training professionals and expanding the care network could make it an effective instrument for reducing mortality from suicide.

## Introduction

Suicide is classically defined as "death resulting directly or indirectly from a positive or negative act of the victim himself, which he knows will produce this result" and is a complex and multifactorial phenomenon.^1^ Among the various risk factors associated with suicide, there is usually presence of a mental disorder and previous suicidal behavior. Hopelessness, impulsivity, access to more lethal means, and family history also play significant roles in determining suicide risk.^2,3^ Conversely, a well-established support network, religious and cultural insertion, good socialization, and access to mental health support services are configured as protective factors against suicidal behaviors, although there are still limited data to support these effects.^3,4^

Because it is a global health problem, several countries are implementing actions related to suicide prevention under World Health Organization (WHO) guidance.^5^ It is known that more than 800,000 people die every year by suicide worldwide, and suicide is already one of the three leading causes of death among young people in Brazil. It is also believed that, although significant, these numbers are underreported, mainly due to social stigma, which can cause many cases to be reported as accidents, for example.^5,6^ When analyzing the data regarding suicide attempts, the situation becomes even more worrying, given the scarcity of data regarding suicidal behavior and the estimate that for each consummated suicide, there are about 20 attempts.^7,8^

As a manifestation of the human experience, suicidal behavior usually produces a broad impact, and the most commonly affected sphere is the social one, which encompasses the family, friends, and acquaintances of the individual who has committed or attempted suicide. From this perspective, grief has profound psychological consequences for survivors bereaved by suicide.^9-11^

Around the world, numerous campaigns have been implemented to reduce the number of deaths by suicide and suicide attempts, as well as to minimize their consequences. Most of these projects have been based on universal strategies, i.e., their target audience is the general population, although actions at the selective level (concerning risk groups) and indicated level (focused on people with suicidal behavior) have also been shown to be important.^12-15^ The few studies that exist on the impacts of suicide prevention campaigns are inconsistent, limited in their designs, or inconclusive, and it is not possible to infer which strategies are most effective or whether actions currently used have helped to address the problem.^8,12,15-17^ It is known, however, that talking about the topic and promoting awareness actions can decrease stigma and reduce anxiety associated with the subject.^8,12^

In Brazil, a law was implemented in 2019^18^ establishing a National Policy for the Prevention of Self-harm and Suicide (Política Nacional de Prevenção da Automutilação e do Suicídio), but even before that, the Life Appreciation Center (Centro de Valorização à Vida [CVV]), in conjunction with the Brazilian Association of Psychiatry (Associação Brasileira de Psiquiatria [ABP]) and the Brazilian Federal Council of Medicine (Conselho Federal de Medicina [CFM]) have been running a national information campaign called Yellow September (YS) (Setembro Amarelo) since 2015.^14^ The campaign promotes actions at various levels, such as walks, lectures, and illumination of public monuments in yellow light to raise awareness about the relevance of the discussion; making free material available online, to raise awareness not only among health professionals, but also within civil society in general. In addition, the campaign includes educational actions with the media aimed to provide guidance and clarify the need to talk about the issue responsibly, due to the possibility of the reverse effect, meaning that the dissemination of data on suicides in an inappropriate way encourages more deaths by suicide. However, contrary to what was expected, but following the trend in other countries, the number of suicides in Brazil does not seem to have decreased.^14,19^

Considering that the YS campaign was implemented at a national level, it constitutes a "natural experiment" that allows this study to analyze the initiative's impact on the number of suicide deaths in the country, taking into account its specificities and convergences with other communicative measures related to suicide.

## Materials and methods

This is an ecological analytical study of the interrupted time series type that analyzes the evolution of suicide rates in Brazil between 2011 and 2019 and its association with the YS campaign. Because of the public nature of the data explored, the study was exempted from ethical approval, according to Brazilian legislation (Resolution 510/2016, issued by the Brazilian National Health Council [Conselho Nacional de Saúde]).

The monthly death count was obtained through secondary data provided by the Mortality Information System (Sistema de Informações Sobre Mortalidade [SIM]), managed by the Information Technology Department of the Brazilian Unified Health System (Departamento de Informática do Sistema Único de Saúde [DATASUS]), using codes X60 to X84 of the International Classification of Diseases (ICD-10), which encompass all causes of death defined as suicide.^20^ We also obtained the monthly numbers of deaths using age group and sex filters to analyze these subgroups.

Demographic data were obtained from the monthly population projections fixed on the 15th of each month and from the annual population projections by sex and age bracket, both provided by the Brazilian Institute of Geography and Statistics (Instituto Brasileiro de Geografia e Estatística [IBGE]).^21^ Since the monthly projections only indicate the overall population, without breakdowns by sex or age groups, it was necessary to calculate the monthly populations of each of these groups by multiplying the national monthly data by the proportions of the subgroups in the annual estimates, thus obtaining the monthly distributions by sex and age groups. These data were used to calculate suicide rates (no. of deaths per 100,000 inhabitants).

In the exploratory analysis, the average suicide rates for the pre-YS, post-YS, and entire periods were calculated, as well as the percentage changes from initial to final rates for these periods.

In an interrupted series study, a given variable is used to determine a historical trend, which is affected by an intervention at a certain point in time. Theoretically, if the intervention had not been applied, the pre-existing historical trend would continue to determine the values, making it possible to compare this counterfactual condition with the post-intervention reality. Compatible with this goal, we performed a segmented interrupted-series regression using a generalized linear Poisson model, ideal for studies in which the variable is a count. Because there was overdispersion in the count data (variance > average), it was necessary to relax the model, switching to a quasi-Poisson model, making it possible to analyze changes in the level and slope of the regression from temporal interventions with adjustment for underlying historical tendencies. To correct for seasonal patterns, four-pair Fourier time-harmonic functions were added to the model. The 95% confidence interval (95%CI) of the model was also calculated. This made it possible to detect any series of occurrences that deviate from an expected curve corrected for historical trends (linear growth) and seasonal trends (harmonic functions).^22^

The ratios between the incidence rates (IR) for the periods after and before the intervention, as well as their respective 95%CI, were estimated by exponentiation of the coefficients of the intervention factor from the different temporal models analyzing the general population, sex subgroups, and age bracket subgroups. The counterfactual condition for each model, i.e., the estimated number of deaths in the absence of the intervention, was calculated using the ratio between the number of deaths predicted by the model and the IR of the intervention.

With regard to the effects of the intervention, two scenarios were studied: a) the influence of the YS campaigns considering an annual effect, with the aggregate intervention factor defined as positive in all months after September 2015 and the annually independent intervention factors as positive only in each appropriate year; and b) the influence of the YS campaigns considering a temporary effect restricted to September, October, November, and December of each year, with the aggregate intervention factor defined as positive in each of these specific months from 2015 onwards and the annually independent intervention factors defined as positive only in the last 4 months of each year.

The null hypothesis that the interventions did not change the historical and seasonal tendency of the evolution of suicide rates in Brazil could be refuted at a significance level of α ≤ 5%. All analyses were performed in Stata v16.0 software.

## Results

From 2011 to 2019, 102,718 suicides were recorded in Brazil. The annual rates were increasing, rising from 4.99 to 6.41 suicides per 100,000 inhabitants, equivalent to an increase of 28.5% over this period. Most of the individuals were male (n = 80,808; 78.7%), with a mortality ratio of 3.86 compared to the female sex. As for age groups, the highest average mortality rate for the period was in the 30 to 59 age group (7.60 per 100,000 inhabitants), while the lowest rate was in the under 14 years age group (0.32 per 100,000 inhabitants). Table 1 shows the average rates by subgroups and their variations over the period.

**Table 1:** Mean suicide rates and their percentage changes from pre-intervention to post-intervention (the first Yellow September [YS] [Setembro Amarelo] was in 2015), by subgroups

Group	2011-2019		Pre-YS: 2011-2014		Post-YS: 2015-2019	
	ASR*	Δ%ASR† (%)	ASR*	Δ%ASR† (%)	ASR*	Δ%ASR† (%)
Country	5.58	28.5	5.17	8.1	5.91	21.0
Male	8.97	28.1	8.31	5.8	9.50	17.0
Female	2.33	30.6	2.16	4.1	2.47	17.9
≤ 14 years	0.32	86.1	0.27	37.9	0.37	44.7
15-29 years	6.03	35.1	5.56	-1.7	6.40	35.1
30-59 years	7.60	19.2	7.23	6.0	7.90	9.9
60-79 years	7.59	17.8	7.05	-0.8	8.03	4.9
≥ 80 years	7.36	-10.8	7.61	5.2	7.16	-17.8

Based on Mortality Information System (Sistema de Informações Sobre Mortalidade [SIM]) data.

* Average annual suicide rate for the period.

† Percentage change in the suicide rate for the period.

### First scenario: analysis of the annual campaign effects after September 2015

**Figure 1:**
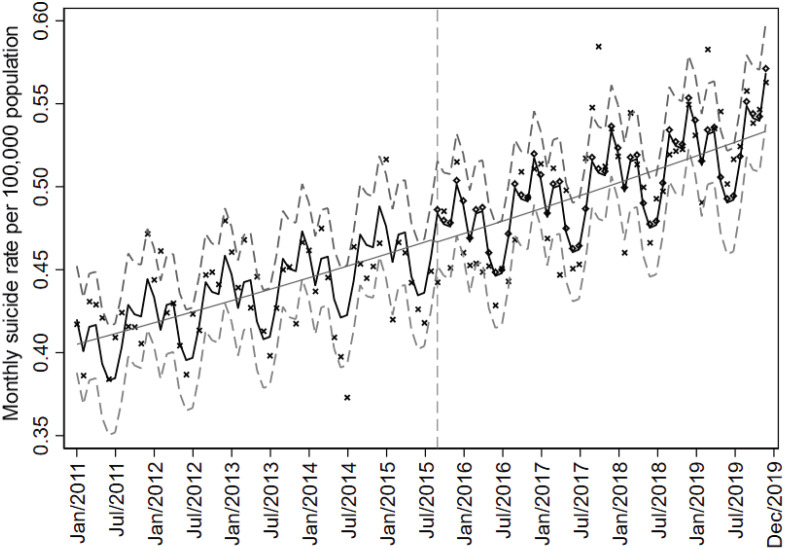
Interrupted time series for monthly suicide rates in Brazil (2011-2019). The intervention is represented by the vertical dashed line, indicating the first Yellow September campaign in 2015. The black line represents the values calculated by Poisson regression with harmonic adjustment for seasonality and historical trend, while the gray dashed lines represent the 95% confidence interval (95%CI) bounds of this model. The solid gray line represents the regression without seasonality adjustment. The observed suicide rate is illustrated by the Xs, while the rate projected by the regression disregarding the intervention is represented by the hollow diamonds. Based on Mortality Information System (Sistema de Informações Sobre Mortalidade [SIM]) data.

Considering the YS campaigns analyzed as a single intervention factor at the national level, we detected a small drop in the level of the regression after the implementation of the first campaign (2015), with a statistically insignificant reduction of 0.47% in the risk of death compared to the counterfactual condition (relative risk [RR] = 0.995; 95%CI 0.959-1.032; p = 0.797), confirming the null hypothesis that this intervention did not modify the historical trend of increasing suicides in Brazil in the period after its implementation (Figure 1). From this hypothesis, subgroup analyses by sex and age group also suggested no significant effect on suicide rates (Table 2).

**Table 2:** Pre-intervention and post-intervention risk ratios considering the annual effects of the campaigns independently and in aggregate, with adjustments for long-term trends and seasonality, from 2015 to 2019

Group	2015		2016		2017		2018		2019		2015-2019	
	RR (95%CI)	p-value	RR (95%CI)	p-value	RR (95%CI)	p-value	RR (95%CI)	p-value	RR (95%CI)	p-value	RR (95%CI)	p-value
Country	0.996 (0.946-1.049)	0.875	0.999 (0.960-1.040)	0.967	1.062 (1.013-1.112)	**0.012**	1.051 (0.996-1.110)	0.067	1.086 (1.020-1.155)	**0.009**	0.995 (0.959-1.032)	0.797
Male	1.004 (0.952-1.059)	0.881	1.005 (0.964-1.047)	0.800	1.060 (1.011-1.113)	**0.017**	1.049 (0.992-1.109)	0.095	1.080 (1.013-1.152)	**0.017**	1.002 (0.966-1.040)	0.897
Female	0.966 (0.876-1.065)	0.484	0.976 (0.906-1.052)	0.527	1.064 (0.976-1.160)	0.158	1.061 (0.960-1.173)	0.246	1.105 (0.985-1.239)	0.088	0.968 (0.907-1.034)	0.344
≤ 14 years	0.860 (0.600-1.232)	0.410	0.936 (0.702-1.247)	0.650	1.057 (0.760-1.471)	0.741	0.896 (0.606-1.324)	0.581	0.945 (0.604-1.478)	0.805	0.952(0.748-1.210)	0.687
15-29 years	0.946 (0.872-1.026)	0.184	1.008 (0.947-1.073)	0.794	1.117 (1.040-1.200)	**0.002**	1.168 (1.074-1.269)	**0.001**	1.333 (1.213-1.465)	**0.001**	0.948 (0.885-1.015)	0.128
30-59 years	1.001 (0.940-1.066)	0.971	0.989 (0.942-1.039)	0.656	1.034 (0.977-1.094)	0.252	1.008 (0.943- 1.077)	0.820	1.020 (0.946-1.101)	0.597	0.998 (0.957-1.042)	0.956
60-79 years	1.069 (0.965-1.184)	0.199	1.041 (0.960-1.128)	0.329	1.105 (1.006-1.214)	**0.037**	1.085 (0.972-1.210)	0.146	1.054 (0.929-1.196)	0.417	1.069 (0.998-1.145)	0.056
≥ 80 years	1.112 (0.886-1.398)	0.357	0.956 (0.799-1.144)	0.627	0.9714 (0.789-1.196)	0.785	0.967 (0.760-1.230)	0.785	0.844 (0.639-1.116)	0.235	0.945 (0.811-1.102)	0.627

Based on Mortality Information System (Sistema de Informações Sobre Mortalidade [SIM]) data.

95%CI = 95% confidence interval; RR = relative risk.

Bold type indicates statistical significance at p < 0.05.

Considering the different campaigns run in each of the last 5 years as independent interventions, it was possible to evaluate the annual impact of each of them, as shown in Table 2. In none of these years was there a significant association between the campaign and the reduction in the death rate. On the contrary: in 2017, there was actually a 6.2% increase in mortality risk, with relevant effects in the male subgroup (p = 0.017) and in the age groups from 15 to 29 years (p = 0.002) and from 60 to 79 years (p = 0.037), although there was no impact in the female subgroup or the other age groups. Similarly, in 2019, there was an 8.6% increase in risk, with a significant impact only among males (p = 0.017) and in the 15 to 29 age group (p < 0.001). It is worth noting that in 2018, even though the risk ratio at the national level was not changed by the intervention, there was a 16.8% increase in suicide risk for the 15 to 29 age group (p < 0.001).

### Second scenario: analysis of temporary effects from September to December only, starting in 2015

Considering only September and the following 3 months in each of the five years of the campaign as a single intervention factor, a statistically insignificant 1.2% increase was observed in the national suicide risk during these periods, even adjusting for seasonality effects (RR = 1.012; 95%CI 0.977-1.048; p = 0.503).

As for the year-specific analyses, there was a statistically significant 6.7% increase in suicide risk in the context of the September campaign and in subsequent months of 2017 (Table 3), with subgroup analyses demonstrating effects only in the male population (p = 0.004) and in the 30 to 59 age group (p = 0.027) and confirming the null hypothesis for females and all other age groups. It is worth noting that the highest monthly suicide rate from 2011 to 2019 was found precisely in this timeframe, at 0.58 suicides/100,000 population in October 2017.

**Table 3:** Pre-intervention and post-intervention risk ratios considering the temporary effect (September to December) of the campaigns independently and in aggregate, with adjustments for long-term trends and seasonality, from 2015 to 2019

Group	2015		2016		2017		2018		2019		2015-2019	
	RR (95%CI)	p-value	RR (95%CI)	p-value	RR (95%CI)	p-value	RR (95%CI)	p-value	RR (95%CI)	p-value	RR (95%CI)	p-value
Country	0.983 (0.933-1.035)	0.518	0.998 (0.948-1.051)	0.961	1.066 (1.013-1.122)	**0.013**	1.004 (0.952-1.059)	0.873	1.018 (0.964-1.074)	0.519	1.012 (0.977 – 1.048)	0.503
Male	0.997 (0.947-1.050)	0.934	1.017 (0.966-1.071)	0.511	1.078 (1.024-1.134)	**0.004**	1.011 (0.959-1.067)	0.667	1.031 (0.976-1.089)	0.263	1.002 (1.002-1.002)	0.160
Female	0.930 (0.844-1.025)	0.147	0.931 (0.844-1.026)	0.150	1.025 (0.932-1.127)	0.604	0.977 (0.886-1.078)	0.656	0.970 (0.877-1.073)	0.564	1.002 (1.002-1.003)	0.258
≤ 14 years	0.936 (0.661-1.326)	0.713	1.124 (0.812-1.556)	0.480	1.219 (0.885-1.679)	0.225	1.112 (0.795-1.555)	0.533	1.057 (0.746-1.497)	0.754	1.083 (0.864-1.359)	0.487
15-29 years	0.910 (0.825-1.002)	0.057	0.963 (0.875-1.060)	0.448	1.079 (0.983-1.185)	0.108	1.070 (0.972-1.178)	0.162	1.145 (1.040-1.262)	**0.006**	1.015 (0.949-1.086)	0.652
30-59 years	1.000 (0.942-1.061)	0.988	1.013 (0.954-1.075)	0.668	1.068 (1.007-1.133)	**0.027**	0.976 (0.917-1.038)	0.444	0.987 (0.927-1.052)	0.706	1.011 (0.970-1.053)	0.590
60-79 years	1.031 (0.932-1.140)	0.545	0.994 (0.898-1.100)	0.916	1.053 (0.953-1.164)	0.304	0.988 (0.891-1.095)	0.821	0.955(0.859-1.062)	0.400	1.009 (0.942-1.080)	0.794
≥ 80 years	1.091 (0.876-1.358)	0.435	0.907 (0.716-1.149)	0.419	0.836 (0.653-1.068)	0.153	0.990 (0.783-1.253)	0.939	0.852(0.662-1.096)	0.214	0.945 (0.811-1.101)	0.472

Based on Mortality Information System (Sistema de Informações Sobre Mortalidade [SIM]) data.

95%CI = 95% confidence interval; RR = relative risk.

Bold type indicates statistical significance at p < 0.05.

Although the 2019 campaign showed no effect on the historical and seasonal trend (p = 0.525) in the national number of deaths, a statistically significant 14.6% increase in suicide risk was detected for the 15-29 years subgroup (p = 0.006), with no effects when analyzing the other age groups or sex subgroups.

There were no significant findings in any of the analyses of the other years or their subgroups, confirming the null hypothesis for all these unmentioned situations.

## Discussion

The descriptive results presented in Table 1 regarding the increase in the number of suicides over recent years are in line with a national trend for this public health problem to worsen.^23,24^ The higher mortality rate among adults and males is consistent with findings in the literature related to the topic.^25^

As shown in both scenarios, the reduction in the number of suicides with the introduction of the campaign in 2015 was statistically insignificant. A similar study that analyzed the prevalence of suicide notifications in Brazil before and after the implementation of the YS^19^ corroborates this finding. In fact, it even presents evidence that suicide rates increased after implementation of the campaign, with emphasis on the months in the second half of the year. In the same vein, an ecological time-series study of suicides in the Brazilian state of Ceará also found no statistically significant difference between years before and after implementation of the campaign.^14^

We had theorized that there would be differences between the campaigns because in each year the campaign's reach and communicative impact on the population would be unique. Therefore, the quality of the campaign could presumably tend towards additive improvement, where each iteration could build on past experiences. However, this was not observed. Moreover, if implementation of the YS was not capable of reducing the number of deaths, neither can it be said that its effect was harmless, given the statistically significant findings indicating an increase in suicides in specific annual campaigns.

In the first scenario, modeling an annual effect, the 2017 and 2019 campaigns were especially problematic, with increases in both general populations and in the male and 15-29-year-old subgroups. In 2018, an effect was also detected in this age group alone. In the second scenario, on the other hand, there was an increase in suicide rates after the 2017 campaign, with an emphasis on males and the 30 to 59 years subgroup. In 2019, there was a negative effect in the 15-to-29-year-old subgroup only.

Despite the above, it is unlikely that the YS campaign was directly responsible for the growth in deaths. Rather, what can be surmised is that its effect was insufficient to reduce suicides during the period, while also failing to maintain a certain level of stability.

These data are in line with literature proposing that campaigns focused solely on publicity in the media generate unsound findings regarding effective reduction of the number of deaths by suicide, since they do not translate into health care provision or modification of psychosocial situations that are risk factors for fatal outcomes.^13,17,26^ However, these actions seem to be important in the sense of increasing the amount of information about the subject in society in general, reducing stigma and increasing demand for services focused on mental health.^15^ Moreover, although campaigns of this type can promote an increase in the demand for specialized help, there is not always an increase in the supply of services, which, in Brazil, are mainly provided by centers for psychosocial care (Centro de Atenção Psicossocial [CAPS]).^17^

Another important point to raise is the hypothesis supported by Table 2, which is that by not changing the rising trend in suicide in Brazil, the YS caused harmful results, which could be explained by the Werther effect.^27^

In this context, campaigns that adopt broad and multi-pronged actions tend to have more promising results, as supported by a systematic review with meta-analysis that found more significant effects from multilevel campaigns.^12^ Take, for example, the research that demonstrated significant decreases in numbers of suicides with the implementation of the Nuremberg Alliance Against Depression (NAD) and the "Zero Suicide" campaigns.^28,29^ These campaigns’ objectives include acting in a permanent way toward: a) training physicians to screen suicidal patients and manage these cases in a systematic and evidence-based manner; b) reducing access to means; c) developing selective and indicated strategies; and d) promoting longitudinal follow-up of patients presenting suicidal behavior. Another systematic review,^30^ while attesting to the effectiveness of withdrawing means, maintaining longitudinal follow-up, and setting up centers focused on emergency care, failed to validate the effectiveness of training physicians and running information campaigns for suicide prevention.

In the context of medical education, the findings of interventional studies suggest that specific training for physicians on suicide risk management, especially in the context of primary care, can generate positive impacts in reducing cases of death by suicide.^31,32^ However, while the 2014 National Curriculum Guidelines (Diretrizes Curriculares Nacionais) (NCGs) contain sections highlighting the importance and mandatory inclusion of topics related to mental health and medical emergencies, only a small proportion of Brazil's Federal medical schools address this topic in their curricula.^33^

From this perspective, the fact that YS is a one-off action that does not emphasize training for physicians and other professionals working in primary health care and emergency room settings may be one reason why the findings of this study were not as expected.^34,35^

For purposes of comparison, suicide mortality rates have decreased globally from 2010 to 2019.^36^ The suicide mortality rate decreased by 4.7% on the American continent when the average suicide rates between 2010-2014 and 2015-2019 are compared, while in the southern cone a comparison between the same periods reveals a 2% decrease.^37,38^ Our study shows a 14.3% increase when comparing the average suicide rates from 2011-2014 and 2015-2019, which means it is unlikely that international or regional tendencies could influence our conclusions regarding the effectiveness of the YS.

When considering the findings and considerations of this study, it is important to point out the following limitations: a) the data were from a secondary database (SIM-DATASUS) and may contain filling errors and underreporting of deaths; b) as this is an ecological study that evaluated the impact of a national campaign, the conclusions are drawn only for the aggregate population and are neither intended nor able to discuss the cases individually; c) although the statistical model controlled for variations due to the rising historical trend and seasonality, other variables potentially correlated with national suicide rates, such as unemployment level and proportion of single people, were not considered.

## Conclusion

This study demonstrated that implementation of the YS project was unable to reduce the historical rising trend in the suicide rate in Brazil. Despite its proposals of disseminating valuable information on mental health, reducing stigma, and encouraging individuals to seek professional assistance, the campaign lacks initiatives rooted in multisectoral actions, such as development of the care network, longitudinal strategies for monitoring risk groups, training, and professional development, which may explain its failure. Development of new programs focused on these questions, in order to improve the campaign, could make it an effective instrument for reducing suicide mortality.
